# Spontaneous and Vaccine-Induced Clearance of Mus Musculus Papillomavirus 1 Infection

**DOI:** 10.1128/JVI.00699-17

**Published:** 2017-07-12

**Authors:** Rosie T. Jiang, Joshua W. Wang, Shiwen Peng, Tsui-Chin Huang, Chenguang Wang, Fabiana Cannella, Yung-Nien Chang, Raphael P. Viscidi, Simon R. A. Best, Chien-Fu Hung, Richard B. S. Roden

**Affiliations:** a Department of Pathology, The Johns Hopkins University, Baltimore, Maryland, USA; b Department of Oncology, The Johns Hopkins University, Baltimore, Maryland, USA; c Department of Gynecology and Obstetrics, The Johns Hopkins University, Baltimore, Maryland, USA; d Department of Pediatrics, The Johns Hopkins University, Baltimore, Maryland, USA; e Department of Otolaryngology-Head and Neck Surgery, The Johns Hopkins University, Baltimore, Maryland, USA; f Graduate Institute of Cancer Biology and Drug Discovery, College of Medical Science and Technology, Taipei Medical University, Taipei, Taiwan, Republic of China; g Istituto Pasteur Italia-Cenci Bolognetti Foundation, Sapienza University, Rome, Italy; h Papivax LLC, Baltimore, Maryland, USA; i Papivax Biotech Inc., Taipei, Taiwan, Republic of China; International Centre for Genetic Engineering and Biotechnology

**Keywords:** DNA vaccines, SKH-1 mice, T cells, mouse model, neutralizing antibodies, papillomavirus

## Abstract

Mus musculus papillomavirus 1 (MmuPV1/MusPV1) induces persistent papillomas in immunodeficient mice but not in common laboratory strains. To facilitate the study of immune control, we sought an outbred and immunocompetent laboratory mouse strain in which persistent papillomas could be established. We found that challenge of SKH1 mice (Crl:SKH1-Hrhr) with MmuPV1 by scarification on their tail resulted in three clinical outcomes: (i) persistent (>2-month) papillomas (∼20%); (ii) transient papillomas that spontaneously regress, typically within 2 months (∼15%); and (iii) no visible papillomas and viral clearance (∼65%). SKH1 mice with persistent papillomas were treated by using a candidate preventive/therapeutic naked-DNA vaccine that expresses human calreticulin (hCRT) fused in frame to MmuPV1 E6 (mE6) and mE7 early proteins and residues 11 to 200 of the late protein L2 (hCRTmE6/mE7/mL2). Three intramuscular DNA vaccinations were delivered biweekly via *in vivo* electroporation, and both humoral and CD8 T cell responses were mapped and measured. Previously persistent papillomas disappeared within 2 months after the final vaccination. Coincident virologic clearance was confirmed by *in situ* hybridization and a failure of disease to recur after CD3 T cell depletion. Vaccination induced strong mE6 and mE7 CD8^+^ T cell responses in all mice, although they were significantly weaker in mice that initially presented with persistent warts than in those that spontaneously cleared their infection. A human papillomavirus 16 (HPV16)-targeted version of the DNA vaccine also induced L2 antibodies and protected mice from vaginal challenge with an HPV16 pseudovirus. Thus, MmuPV1 challenge of SKH1 mice is a promising model of spontaneous and immunotherapy-directed clearances of HPV-related disease.

**IMPORTANCE** High-risk-type human papillomaviruses (hrHPVs) cause 5% of all cancer cases worldwide, notably cervical, anogenital, and oropharyngeal cancers. Since preventative HPV vaccines have not been widely used in many countries and do not impact existing infections, there is considerable interest in the development of therapeutic vaccines to address existing disease and infections. The strict tropism of HPV requires the use of animal papillomavirus models for therapeutic vaccine development. However, MmuPV1 failed to grow in common laboratory strains of mice with an intact immune system. We show that MmuPV1 challenge of the outbred immunocompetent SKH1 strain produces both transient and persistent papillomas and that vaccination of the mice with a DNA expressing an MmuPV1 E6E7L2 fusion with calreticulin can rapidly clear persistent papillomas. Furthermore, an HPV16-targeted version of the DNA can protect against vaginal challenge with HPV16, suggesting the promise of this approach to both prevent and treat papillomavirus-related disease.

## INTRODUCTION

There are over 200 known human papillomaviruses (HPVs) (1) that infect skin or mucosal epithelia. While infections with most skin-associated types are clinically inconsequential and/or self-limiting, subsets of mucosal HPV genotypes persist and produce a variety of diseases, including anogenital warts; benign warts; as well as anal, vaginal, penile, oropharyngeal, and cervical carcinomas (2–5). Genital HPV is the most common sexually transmitted disease (6, 7), and cervical cancer is the fourth leading cause of cancer deaths in women worldwide, 50 to 60% of which are driven by HPV16 (6, 8, 9). HPV16 is predominant (>90%) in oropharyngeal cancer and other anogenital cancers (6, 7, 10, 11).

Mucosal HPVs have been categorized based on their oncogenic potential as low-risk HPV (lrHPV) types (typified by HPV6 and -11), which are associated with benign genital warts, and a dozen high-risk HPV (hrHPV) types (exemplified by HPV16, -18, -31, -33, -45, -52, and -58), which are associated with malignant disease and have been targeted for vaccine development (7, 12). The three licensed preventive HPV vaccines, Cervarix (HPV16 and -18; GlaxoSmithKline), Gardasil (HPV6, -11, -16, and -18; Merck & Co.), and Gardasil-9 (HPV6, -11, -16, -18, -31, -33, -45, -52, and -58), are composed of virus-like particles (VLPs) formed from the major capsid protein L1 of the indicated genotypes (13–18). Although these vaccines provide robust protection against new infections of the targeted HPV types, they are not typically active against other types (19), and their high cost remains a barrier to global distribution (20). As an alternative to increasingly multivalent L1 VLP vaccines, vaccination with the linear and broadly conserved protective epitopes within the first 200 residues of the L2 capsid antigen shows promise to address issues of type-restricted protection (21). However, neither L1- nor L2-based vaccines are effective in clearing existing infections.

Naked-DNA-based therapeutic vaccination targeting early antigens has shown considerable promise for the treatment of CIN2/3 (21–24). In a recent phase I study using the DNA vaccine pNGVL4a-hCRTE7(detox) administered intradermally (i.d.), intramuscularly (i.m.), or intralesionally, 8/27 patients with HPV16-positive (HPV16^+^) cervical intraepithelial neoplasia grade 2/3 (CIN2/3) exhibited histological regression (25). To enhance its immunogenicity by coupling it to a “danger-associated molecular pattern” (DAMP) (26), this DNA vaccine expresses human calreticulin (CRT) fused in frame to HPV16 *E7* doubly mutated to eliminate oncogenicity (detox) (27). Since HPV16 E6 may be a dominant antigen in patients, and L2 antibodies are protective, a DNA vaccine, pNGVL4a-hCRTE6E7L2, which also targets HPV16 E6 and residues 11 to 200 of L2, was developed (28). Vaccination of mice with pNGVL4a-hCRTE6E7L2, but not the vector alone, generated both L2-specific neutralizing serum antibodies and E6/E7-specific antitumor immunity in mice, suggesting its potential as a prophylactic/therapeutic HPV vaccine (28, 29). Finally, the administration of the DNA by *in vivo* electroporation proved superior to conventional i.m. administration or i.d. ballistic delivery (30, 31).

The development of therapeutic vaccines has been reliant on animal papilloma models in rabbits, dogs, cows, and Mastomys coucha, for which immunology studies are more challenging than for laboratory mice (32–34), or HPV16 E6/E7-expressing syngeneic mouse tumor models, which do not fully replicate disease, e.g., TC-1 (28) or C3 (35). Since Mus musculus papillomavirus 1 (MmuPV1) produces persistent disease only in immunocompromised laboratory mice (36, 37), our goal here was to develop an MmuPV1 model of persistent papillomas in an outbred laboratory mouse strain with an intact immune system for the study of both spontaneous and vaccine-driven viral clearances.

## RESULTS

### Development of warts in SKH-1 mice after MmuPV1 challenge.

**FIG 1:**
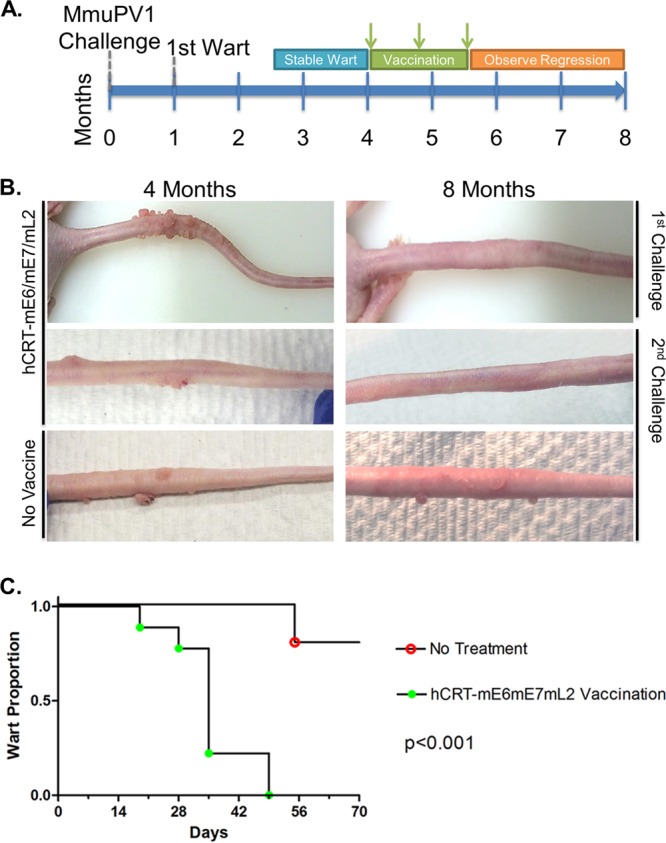
Development of MmuPV1 papillomas in immunocompetent, outbred SKH-1 mice. (A) SKH-1 mice were challenged on the tail with 40 μl of a previously harvested papilloma crude lysate from athymic nude mice. Initial warts were apparent ∼1 month after the initial challenge. Warts were monitored for 4 months postchallenge. Mice that developed papillomas which then self-resolved within these 4 months were defined as having “transient” disease. Mice that developed papillomas that were maintained or continued to progress in months 2.5 to 4 were considered to have “persistent” disease. The remaining mice never developed warts within 4 months of challenge. (B) Of 20 mice from the first challenge, 4 mice developed persistent papillomas and were subsequently treated with 15 μg/dose/mouse of the DNA vaccine hCRT-mE6mE7mL2 and electroporation three times biweekly (the tail of a representative animal is shown in the first row, which was photographed at 4 months [left] or 8 months [right] postchallenge). Mice, regardless of persistent disease burden, were able to fully resolve all clinically apparent papillomas within 2.5 months after the final vaccination. For the second challenge, an additional 95 SKH-1 mice were treated on the tail with MmuPV1. Sixteen mice developed persistent papillomas and were divided into (i) hCRT-mE6mE7mL2 treatment (a representative animal is shown in the second row of panel B) and (ii) no-treatment (a representative animal is shown in the bottom row of panel B) groups. (C) Vaccinated mice were able to clear papillomas within 1.5 months of the final vaccination, while all mice in the no-treatment group maintained their warts. Mice were observed for an additional month. Two of the no-treatment mice self-resolved. The remaining 8/10 mice continued to develop papillomas.

Previous studies in hairless S/RV/Cvi-ba/ba mice produced only transient disease after MmuPV1 challenge (38). No papillomas were seen in FVB/NCr, BALB/cAnNCr, DBA/2NCr, A/JCr, C57BL/6NCr (C57BL/6), 129S6/SvEv, C3H/HeJCr, and outbred Cr:ORL SENCAR (SENCAR) mice after MmuPV1 challenge (39). Interestingly, when mice were treated with cyclosporine, the same strains developed papillomas, and this partially correlated with the previously reported strain-dependent susceptibility to chemical-induced papillomatosis (39). For initial testing, we explored mice of the outbred and immunocompetent strain SKH-1 Elite, which are unpigmented and hairless because of the hypomorphic *hairless* (*Hr*) allele and commonly used to study chemically induced papillomatosis, wound healing, and skin carcinogenesis. Although inbred HRS/J mice, which also carry *Hr*, have limited splenic CD4^+^ T cell function (40), SKH-1 mice have been shown to have antibody and T cell (CD4^+^ and CD8^+^) responses similar to those of C57BL/6 mice (41, 42). SKH-1 mice (10 males and 9 females) were challenged with MmuPV1 virions. Nine of 19 SKH-1 mice (4 females and 5 males) developed clinically apparent warts within 6 weeks postchallenge (Fig. 1A). The timing of wart development was consistent with that in athymic nude mice despite SKH-1 mice having intact CD4^+^ and CD8^+^ T cell function. The papilloma burdens varied between mice from clusters to single warts. The papillomas on 5 of these 9 mice regressed in the following 2 months, leaving 4 mice with persistent warts, half of which continued to grow and spread (Fig. 1B, row 1). Thus, challenge of SKH-1 mice resulted in three distinct clinical outcomes: (i) persistent (>2 months) papillomas (*n* = 4), (ii) transient papillomas that spontaneously regress (typically within 2 months; *n* = 5), and (iii) never any visible papillomas after challenge (*n* = 10).

### Therapeutic treatment of warts by DNA vaccination and electroporation.

DNA vaccines expressing human calreticulin (hCRT)-fused HPV16 E6 and E7 are capable of generating HPV16-specific CD8^+^ T cells and potent antitumor immunity in mice (28, 43). Therefore, we generated a DNA vaccine that expresses hCRT fused with MmuPV1 E6, E7, and L2 (amino acids [aa] 11 to 200) (hCRT-mE6mE7mL2) to assess whether the persistent papillomas in SKH-1 mice could be treated by vaccination. Previous studies indicate that *in vivo* electroporation greatly enhances DNA delivery (44–46), and therefore, the 4 SKH-1 mice with persistent papillomas were vaccinated three times biweekly with hCRT-mE6mE7mL2 DNA by *in vivo* electroporation. These mice were monitored for a further 10 weeks for regression. Warts on 3 of 4 mice regressed within 6 weeks, but one mouse with the greatest papilloma burden needed an additional 4 weeks for complete regression. A second study was performed with 95 SKH-1 mice to control for spontaneous regression in persistent papillomas. Of the 95 mice challenged with MmuPV1, 13 exhibited transient papillomas, and 19 mice developed persistent papillomas. The 19 mice with persistent papillomas were randomized into a treatment group of 9 mice to be vaccinated three times biweekly with hCRT-mE6mE7mL2 DNA and a control group of 10 mice with no treatment. Mice previously treated with the vector plasmid and electroporation exhibited no induction of E6- and E7-specific CD8^+^ T cell responses or antitumor immunity (28, 30), and therefore, untreated mice were used as a control. The mice were observed for a total of 10 weeks, and all 9 vaccinated mice cleared clinically observable warts within 7 weeks postvaccination (Fig. 1B, rows 1 and 2), whereas in the untreated group, most mice retained warts (Fig. 1B, row 3). Of the untreated mice, 2 mice spontaneously regressed at 8 weeks, whereas the 8 other mice retained warts that continued to progress, a significant difference (*P* < 0.0001, by a log rank test) compared to the vaccinated group (Fig. 1C).

### Clearance of MmuPV1 papillomas after vaccination is associated with an MmuPV1-specific CD8 T cell response.

**FIG 2:**
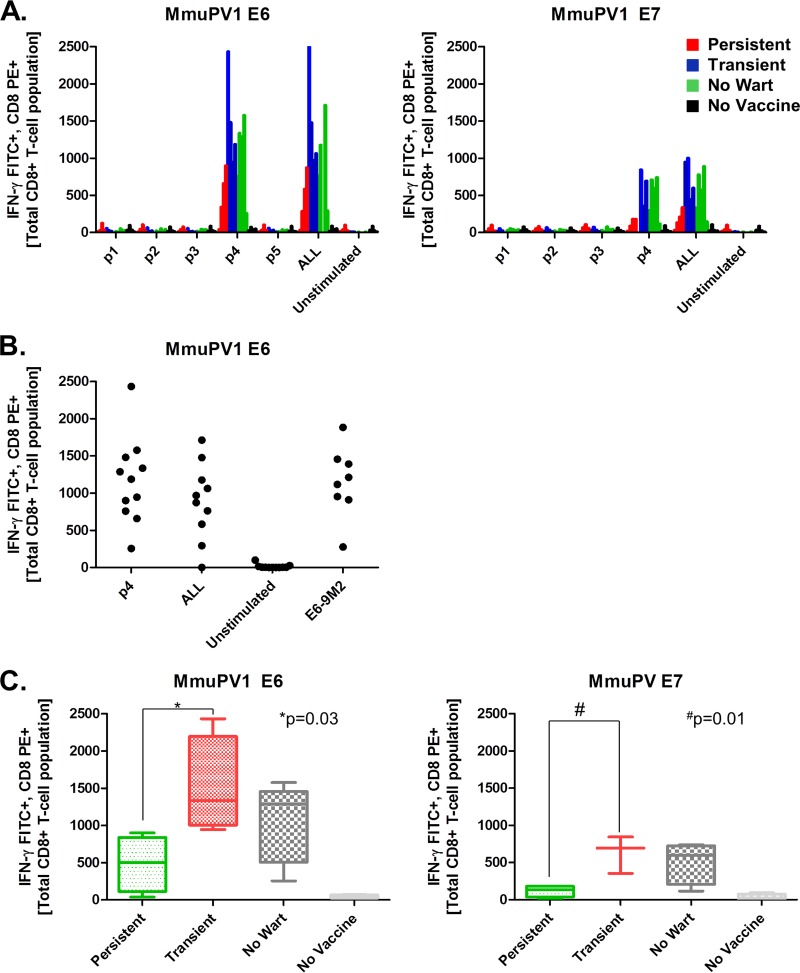
Intracellular cytokine staining with flow cytometry analysis reveals an immunodominant MmuPV1 E6 CD8 T cell response. (A) SKH-1 mice that exhibited persistent or transient warts or never developed MmuPV1 warts and were then vaccinated with a previously described regimen of the hCRT-mE6mE7mL2 vaccine, and their splenocytes were harvested, as were those from nonvaccinated control mice. The graph depicts flow cytometric analysis of splenocytes stained for gamma interferon and CD8 and stimulated with MmuPV1 E6 or E7 peptide library pools. All mice, regardless of disease status and despite the outbred nature of the strain, showed stimulation corresponding to mE6 amino acids 89 to 104 and mE7 amino acids 76 to 95. (B) Splenocytes were stimulated with individual 9-mer peptides corresponding to the previously identified dominant pooled peptides. The dominant mE6 CD8^+^ T cell epitope was identified as KNIVFVTVR, whereas we were unable to define a dominant MmuPV1 E7 CD8 T cell epitope. (C) When stimulated CD8^+^ T cell signals were separated by disease status, mice with persistent papillomas prevaccination were not able to generate MmuPV1 E6 and E7 CD8 T cell responses to DNA vaccination as high as those with regressed papillomas. *P* values were based on Student's *t* test without multiplicity adjustment.

To characterize the CD8^+^ T cell epitopes induced by vaccination, splenocytes were harvested 3 months after the final vaccination and stimulated by using 20-mer overlapping peptide pools that together encompassed MmuPV1 E6 and E7. A strong MmuPV1 E6 CD8^+^ T cell response to amino acids 75 to 115 (p4) and a weaker MmuPV1 E7 CD8^+^ T cell response to amino acids 75 to 110 (p4) were identified in all hCRT-mE6/mE7/mL2-vaccinated mice (Fig. 2A). Using 9-mer peptides overlapping by 1 amino acid, the immunodominant epitope of MmuPV1 E6 was localized at amino acids 90 to 99 (KNIVFVTVR) in all mice (Fig. 2B). This was surprising given that SKH-1 mice are outbred, perhaps suggesting that it is a promiscuous T cell epitope. Indeed, the same 9-mer was previously identified in hCRT-mE6/mE7/mL2-vaccinated C57BL/6 mice but not in BALB/c mice (37). Although overlapping 9-mers for MmuPV1 E7 were developed, no single dominant epitope was identified within residues 76 to 95.

Using Student's *t* test, we compared the MmuPV1 E6 CD8^+^ T cell responses after three vaccinations with hCRT-mE6mE7mL2 DNA in three groups of mice: those that had persistent warts prevaccination, those that had transient warts (that had cleared prior to vaccination), or those that never developed clinically apparent warts. Interestingly, mice with persistent warts at the initiation of vaccination mounted a significantly weaker (without multiplicity adjustment) MmuPV1-specific CD8^+^ T cell response for both E6 (*P* = 0.03) and E7 (*P* = 0.01) antigens than did the group of mice that had spontaneously cleared their warts prior to vaccination (Fig. 2C). The mice who never developed warts exhibited a response similar to that of the latter group. Thus, the persistence of warts may reflect a generally weaker ability to generate MmuPV1-specific CD8^+^ T cell responses.

### Serum antibody response to MmuPV1 challenge and vaccination.

**FIG 3:**
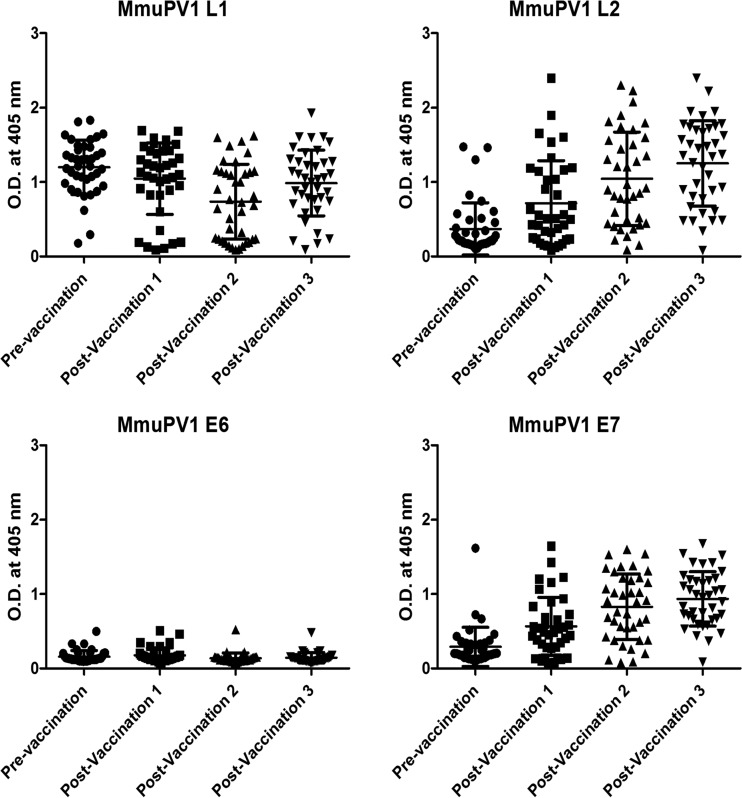
Serum antibody response induced by DNA vaccination. MmuPV1-challenged SKH-1 mice were subsequently vaccinated with 15 μg/dose/mouse of the DNA vaccine hCRT-mE6mE7mL2 and treated with electroporation three times biweekly. Sera were collected 2 weeks later and assessed by an ELISA for serum antibody levels of the MmuPV1 proteins L1, L2, E6, and E7. Mice showed a consistently elevated mL1 VLP antibody response, presumably due to the initial MmuPV1 challenge, since naive animals lacked L1 VLP antibody. Regardless of the prevaccination papilloma status, robust L2 and E7 antibody responses were observed but with a weak E6 response. O.D., optical density.

At 16 weeks post-MmuPV1 challenge, all SKH-1 mice mounted a serum antibody response against MmuPV1 L1 VLPs that was absent from naive mice. The L1 VLP-specific antibody response was thus due to the initial challenge, although these responses were similar regardless of whether warts developed, persisted, or never developed. As expected, the L1 VLP-specific antibody responses before and after vaccination with hCRT-mE6mE7mL2 DNA did not change significantly (Fig. 3). No serum antibody response to E6, E7, or L2 was seen in naive animals, although in a minority of mice, responses were observed after MmuPV1 challenge, as seen in HPV-infected patients (47). We cannot eliminate the possibility that the inoculum rather than active infection triggered these sporadic antibody responses. To determine serum antibody responses to the vaccine antigens, serum collected either before or 1 week after each vaccination was tested by an enzyme-linked immunosorbent assay (ELISA) for reactivity against MmuPV1 E6, E7, or L2 (Fig. 3). In general, SKH-1 mice, regardless of their clinical response to MmuPV1 challenge, mounted robust serum IgG responses to both MmuPV1 E7 and L2 but not to MmuPV1 E6, which showed no clear differences by clinical outcome (data not shown).

### Assessment of virologic clearance after DNA vaccination of mice with persistent warts.

**FIG 4:**
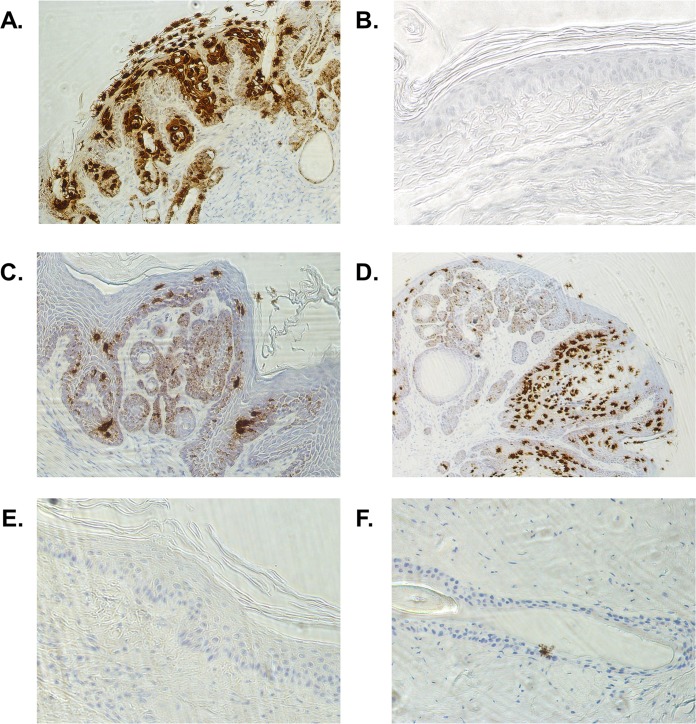
DNA vaccination clears MmuPV1 E6/E7 mRNA. FFPE tail sections from MmuPV1-challenged SKH-1 mice were assessed for MmuPV1 E6/E7 mRNA levels by using RNA CISH. (A and B) Athymic nude mice bearing warts showed strong signals (A), while uninfected mice were free of signals (B). (C and D) Untreated persistent papillomas show strong mE6/mE7 mRNA levels (8/10 mice). (E and F) Treatment of persistent disease in SKH-1 mice by DNA vaccination with hCRT-mE6mE7mL2 elicits complete clearance of MmuPV1 E6/E7 mRNA (9/10 mice). However, 1/10 mice showed 1 cell that had an MmuPV1 E6/E7 mRNA CISH signal.

As previously shown, subclinical infection can be detected in formalin-fixed, paraffin-embedded (FFPE) mouse tail cross sections by using a highly sensitive chromogenic RNA *in situ* hybridization (CISH) technique with a probe against full-length E6/E7 mRNA (not shown) (37). High levels of signal were found in tail epithelia of infected athymic nude mice (Fig. 4A), whereas a signal was absent in all uninfected tails (Fig. 4B). For MmuPV1 E6/E7 mRNA-negative samples, RNA preservation was confirmed by ubiquitin probes and demonstrated a high signal (not shown). Similarly, high levels of the MmuPV1 *E6/E7*-specific signal were detected in 10 nonvaccinated SKH-1 mice bearing warts (Fig. 4C and D). Conversely, 9/10 vaccinated SKH-1 mice that cleared their previously persistent warts lacked an MmuPV1 *E6/E7*-specific signal, and the single remaining mouse exhibited only one stained cell (Fig. 4E and F).

The MmuPV1 *E6/E7*-targeted RNA *in situ* hybridization (ISH) technique appears to detect not only mRNA but also viral DNA, and thus, it is a highly sensitive system with which to detect the presence of MmuPV1 infection. Staining is strong in the upper levels of the papilloma as a large focal nuclear signal, which is consistent with the staining of viral genomes, whereas the fine-dotted signal in the lower epithelium is consistent with the *E6/E7*mRNA signal. Indeed, when treated with RNase A at room temperature for 30 min, the nuclear stain remains in the upper layers, whereas the finely grained staining toward the base of the epithelium is mostly removed (not shown).

### Clearance of subclinical infections.

**FIG 5:**
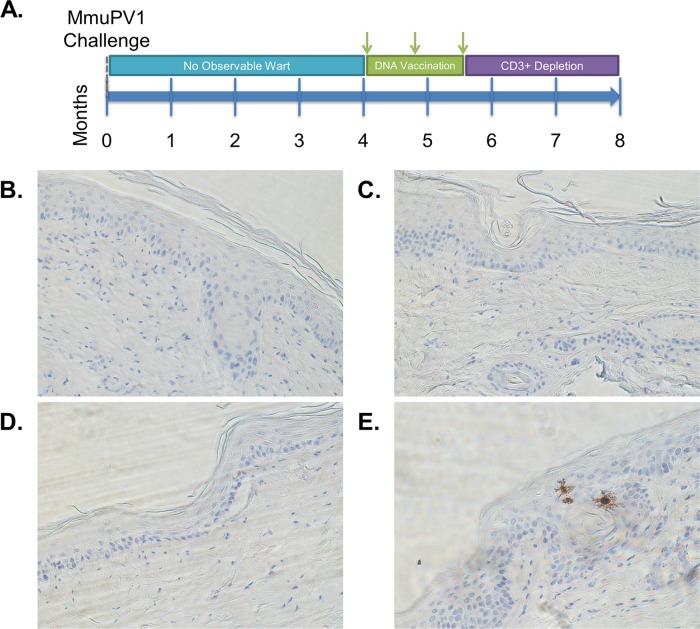
Potential subclinical MmuPV1 reservoir. (A) MmuPV1-challenged SKH-1 mice that never developed papillomas were divided into a no-treatment group and a group vaccinated three times with 15 μg of the DNA vaccine hCRT-mE6mE7mL2. Following 6 weeks of vaccination or no treatment, all mice were depleted of T cells with anti-CD3 antibody. (B to D) RNA CISH shows that 5/5 mice treated with DNA vaccination completely cleared MmuPV1 E6/E7 mRNA (B and C), whereas only 4/5 mice without treatment cleared MmuPV1 E6/E7 mRNA (D). (E) The remaining mouse with no treatment showed a small cluster of MmuPV1 E6/E7 mRNA.

Following challenge, the majority of SKH-1 mice never develop visible warts. However, a subclinical reservoir of MmuPV1 might remain (37). Thus, 10 SKH-1 mice that never developed warts after MmuPV1 infection were divided into two groups: 5 were treated three times biweekly with DNA vaccination with hCRT-mE6mE7mL2 and electroporation, and 5 received no treatment. Two weeks after the final vaccination, all 10 mice were depleted of T cells by weekly CD3-specific monoclonal antibody injection sustained over 6 weeks (Fig. 5A). None of the mice developed papillomas or had an MmuPV1-specific signal (Fig. 5B and C). Only 1 of 5 mice in the untreated cohort showed a single small cluster of MmuPV1-infected cells, suggesting the possibility of lingering subclinical infection that was partially relieved by T cell depletion but failed to produce a visible papilloma (Fig. 5D and E).

### pNGVL4a-hCRTE6E7L2 DNA vaccination protects against vaginal HPV16 challenge.

**FIG 6:**
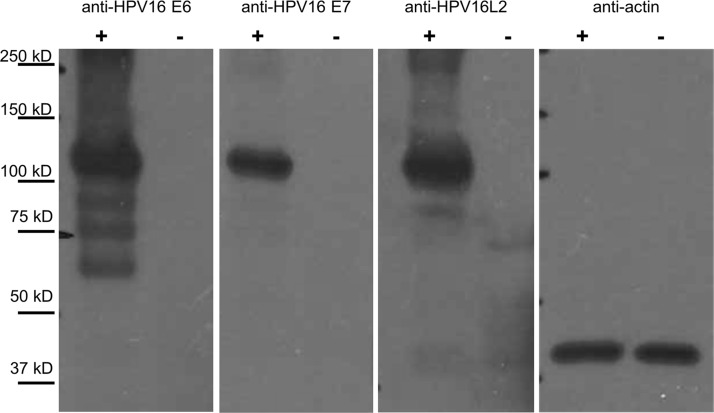
Confirmation of HPV16 E6, E7, and L2 protein expression by the hCRT-HPV16E6E7L2 vaccine. 293TT cells were transiently transfected by using the clinical-grade DNA vaccine hCRT-HPV16E6E7L2, harvested, and probed with monoclonal antibodies specific for HPV16 E6, E7, or L2 by Western blot analysis. Transfected cells (+), but not control cells (−), were reactive to antibodies against all three HPV16 antigens (E6, E7, and L2), with a band size consistent with that of the human calreticulin fusion protein.

**FIG 7:**
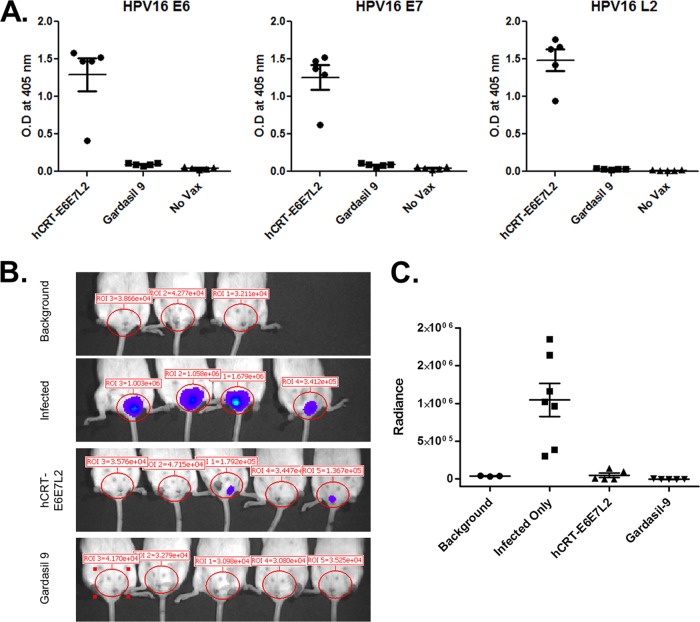
DNA vaccination provides preventative protection against HPV16 infection. BALB/cJ mice were vaccinated three times biweekly with either clinical-grade hCRT-E6/E7/L2 (15 μg/mouse) or Gardasil-9 (1/10 the human dose) and subsequently challenged with HPV16 PsV carrying the luciferase reporter gene. (A) Serum antibody levels from hCRT-E6/E7/L2-vaccinated mice were assayed by an ELISA and showed similar responses against E6, E7, and L2. (B) Efficacy of protection against HPV16 PsV was measured by bioluminescence imaging. The DNA vaccine hCRT-E6/E7/L2 showed complete protection for 3/5 mice, and Gardasil-9 showed compete protection for 5/5 mice. (C) There were statistically no difference (*P* = 0.12) in infection levels between hCRT-E6/E7/L2 and Gardasil-9 treatments; both treatments showed significant protection compared to the challenge group that received no treatment (Gardasil-9, *P* = 0.003; hCRT-E6E7L2, *P* = 0.004).

We generated a pNGVL4a-hCRTE6E7L2 DNA that targets HPV16 E6, E7, and residues 11 to 200 of L2, and it was produced in compliance with good manufacturing practices (cGMP) (31). After transfection into 293TT cells, the DNA vaccine was verified to express the HPV16 E6, E7, and L2 proteins by Western blotting with monoclonal antibodies against each antigen (Fig. 6). In order to test whether DNA vaccination can elicit L2 antibody-mediated protection, BALB/cJ mice were vaccinated three times biweekly using *in vivo* electroporation to deliver the HPV16-targeted version of pNGVL4a-hCRTE6E7L2 DNA. Additional groups (*n* = 5) of mice were vaccinated with Gardasil-9 or left unvaccinated. DNA vaccination induced serum antibodies for all three vaccine antigens HPV16 E6, E7, and L2, as determined by an ELISA (Fig. 7A). Nineteen days after the final immunization, all groups of mice were challenged intravaginally with an HPV16 pseudovirus (PsV) that delivers a luciferase reporter. Three days after challenge, vaginal infection was measured by bioluminescence imaging. Vaccination with Gardasil-9 provided complete protection from experimental challenge, as the bioluminescent signal was not significantly different from that for 3 unchallenged mice. DNA vaccination protected 3 of 5 mice similarly to Gardasil-9, but a weak signal was detected in two mice (Fig. 7B), although overall protection was not significantly different (*P* = 0.12) between these groups (Fig. 7C).

## DISCUSSION

MmuPV1 challenge of mice of the immunocompetent and outbred SKH-1 strain shows considerable promise as a model of HPV disease in humans. Most genital hrHPV infections are asymptomatic and clear within 6 months to a year, but a subset of cases persist and can progress. Likewise, upon MmuPV1 challenge of SKH-1 mice, ∼20% develop persistent papillomas, and ∼15% show transient disease, but most never develop visible papillomas. Thus far, persistent MmuPV1-related papillomas have remained benign. For the study of therapy, for example, it would be beneficial to have a higher proportion of animals that develop persistent papillomas after challenge. Challenge of FVB/NJ mice with MmuPV1 in concert with exposure to UVB radiation, which is associated with systemic immune suppression, was recently shown to produce persistent papillomas (48). In addition to the productive regions of these stable papillomas, multiple areas of atypical squamous hyperplasia were observed, with focal regions consistent with squamous cell carcinoma, including invasion extending into follicular structures deep within the dermis. Notably, SKH-1 mice are very susceptible to UV radiation, which has been used to induce nonmelanoma skin cancer in this strain (49).

MmuPV1-challenged SKH-1 mice with stable papillomas provide a useful model with which to test the potential of a candidate therapeutic vaccine because no immunosuppression is used. After vaccination with the naked DNA hCRT-mE6mE7mL2 via *in vivo* electroporation three times biweekly, SKH-1 mice developed a strong MmuPV1 E6/E7-specific CD8^+^ T cell immune response as well as antibody responses to L2. This regimen was sufficient to drive the complete clearance of these otherwise stable papillomas. We note that in a phase I study of the related vaccine pNGVL4a-hCRTE7(detox) in women with HPV16^+^ CIN2/3, only 8/27 women demonstrated histological clearance postvaccination (25). In our murine study, the vaccine included additional viral antigens (E6 and L2) to broaden the targets of cellular immunity and elicit neutralizing antibody. E6 was included because it may be the dominant antigen in patients (50). L2 was included as a candidate protective antigen (32, 51). In addition, here the DNA was administered by *in vivo* electroporation, as previous studies in mice showed that it greatly enhances both the efficiency of transduction and the resultant immune response over conventional i.m. delivery (45, 46, 52–55). Furthermore, data from previous studies suggest that the difference is even more profound in primates and especially humans (56). Indeed, both of the recent promising trials in CIN2/3 patients with VGX-3100 and GX-188E DNA vaccines, which target HPV16/18 E6/E7, used *in vivo* electroporation (21–23, 57).

L2 is expressed in the upper layers of productive lesions, and therefore, it may also represent a potential target of cellular immunity (58). However, L2 is not detectable in the basal epithelia that harbor the virus and is thus not considered an appropriate target, especially in more progressed lesions (59). L2 residues 11 to 200 contain protective epitopes, and vaccination with DNA expressing this region (in either MmuPV1- or HPV16-derived constructs) induced an L2-specifc antibody response (33, 60). Such a response may be beneficial in preventing new infection/reinfection and/or the spread of infection within the host. Indeed, Vinzon et al. showed that L1 VLP vaccination induced neutralizing antibodies and limited both preexisting infection and the development of skin cancer in the Mastomys natalensis papillomavirus 1 (MnPV1) model (61). The prophylactic potential of hCRT-E6E7L2 vaccination was demonstrated by the protection of BALB/cJ mice against vaginal challenge using an HPV16 PsV carrying a luciferase reporter. Mice were strongly protected, but 2/5 mice showed a marginal bioluminescent signal upon HPV16 PsV challenge, suggesting incomplete protection, whereas complete protection was observed with Gardasil-9 vaccination, consistent with the much lower neutralizing antibody titers induced by L2. However, this model does not measure the protective efficacy of the E6/E7 T cell response that would also be elicited by hCRT-E6E7L2 vaccination. While linkage with hCRT boosts cellular immune responses, it had little benefit for HPV-specific humoral responses (28). However, hCRT-E6E7L2 DNA-priming vaccination followed by a protein-based vaccine boost (e.g., with the HPV16 L2E7E6 fusion protein TA-CIN) is a promising combination (62).

The DNA vector pNGVL4a and the E7(detox) gene have been used safely in 3 clinical studies involving pNGVL4a-Sig/E7(detox)/HSP70 (63, 64) as well as one study with pNGVL4a-CRT/E7(detox) (25). Both components are used in the HPV16-targeted construct pNGVL4a-CRTE6E7L2 used here (65). Its E6(detox) gene contains multiple mutations to disrupt oncogenic activity by targeting key cysteine residues in the two zinc finger domains via mutations of C63 and C106, both of which are mutated to glycine. This approach was previously used in a vaccine construct and shown to eliminate the ability to trigger the degradation of p53 (65). In another study, mutation of either cysteine residue was shown to abolish the immortalization activity of HPV16 E6 (66). Likewise, cells transduced with E6 containing either a C63G or C106G mutation retained normal levels of p53, whereas p53 was almost undetectable in cells expressing wild-type E6 (66). The C63G mutation also serves to knock out the activation of telomerase by E6 (67). To further eliminate potential E6 activity from its second zinc finger motif, the region spanning positions 139 to 144, containing the last two cysteine residues in E6, was also deleted, and the stop codon was deleted for direct fusion to HPV16 E7. HPV16 E7(detox) (25, 63, 64) contains mutations C24G and E26G that eliminate E7's transforming function and binding to the retinoblastoma protein (pRB) (68, 69). In addition, a C91G mutation is included to destroy E7's single zinc finger in conserved region 3 (CR3) (65), which alone eliminates the immortalizing activity of E7 as well as binding to histone deacetylase (HDAC), c-jun, and BRCA1 (70–73). The stop codon is removed from E7 to permit fusion with residues 11 to 200 of HPV16 L2, which is not a viral oncogene (74). Efficient expression of the viral proteins, notably L2, is provided by codon optimization, and a stop codon is added at the 3′ end (75, 76).

While SKH-1 mice with persistent papillomas were able to completely clear their warts after vaccination with hCRT-mE6mE7mL2 DNA, their E6-specific CD8 T cell response was significantly lower after vaccination than the responses to hCRT-mE6mE7mL2 DNA seen in mice that cleared their papillomas or never developed papillomas prior to vaccination. In addition, we previously showed that the adoptive transfer of E6-specific T cells cleared MmuPV1 infection. These findings suggest that the ability to generate a robust E6-specific MmuPV1 CD8^+^ T cell response may correlate with the clinical outcome after MmuPV1 challenge. Notably, systemic DNA vaccination was able to clear skin lesions, demonstrating that local vaccination is not required and that the T cells can home to the lesion site in the epithelium.

The onset of warts and the prevalence of nonmelanoma skin cancer in organ transplant patients suggest that patients carry reservoirs of subclinical or “latent” papillomavirus infection that can be reactivated by iatrogenic immune suppression (77–79). To test whether mice that never developed warts upon initial MmuPV1 challenge harbor such a reservoir, these mice were depleted of T cells. One of five mice showed a cluster of three cells expressing an MmuPV1 E6/E7 signal, demonstrating the potential for such a reservoir to exist, but none of the immune-suppressed mice developed clinically apparent warts. Although it is possible that these cells result from a new infection from the mouse environment, the neutralizing L1-specific antibody response that occurs upon MmuPV1 challenge likely protects against reinfection and suppresses spread upon the reactivation of this virus. Reactivation of the virus to develop a clinically apparent wart may require immunosuppression beyond 6 weeks. Typically, warts develop 1 to 2 months after MmuPV1 challenge, although they arise from a large viral challenge and extensive wound healing. While we did not previously see reactivation in extended immunosuppression studies with BALB/c mice, this has been seen in the cottontail rabbit papillomavirus (CRPV)/rabbit model (77).

In summary, we have established a new immunocompetent and outbred mouse model of papillomavirus infection that mimics clinical outcomes seen in patients with genital HPV infection. While we studied skin disease here, MmuPV1 also replicates at oral, anal, and vaginal sites, suggesting how this model might be further improved (80). Using mice with persistent benign MmuPV1 papillomas, we demonstrate immune-directed clearance through vaccination with a naked DNA, hCRT-mE6mE7mL2, administered with electroporation. In addition, prophylactic vaccination of mice with clinical-grade hCRT-E6E7L2 targeting HPV16 protected against vaginal challenge with an HPV16 PsV, suggesting the potential to prevent the spread of infection within and between patients in concert with therapeutic activity.

## MATERIALS AND METHODS

### Mice and ethics statement.

All animal studies were carried out in accordance with the recommendations in the *Guide for the Care and Use of Laboratory Animals* (81) and with the prior approval of the Animal Care and Use Committee of The Johns Hopkins University (MO12M223). Four- to five-week-old male and female SKH-1 Elite mice (Crl:SKH1-Hr^hr^) and athymic nude mice [Crl:NU(NCr)-*Foxn1*^*nu*^] were obtained from Charles River Inc. BALB/cJ mice (strain code 000651) were obtained from The Jackson Laboratory.

### MmuPV1 production and infection challenge.

As previously described (82), athymic nude mice were used to propagate MmuPV1 viruses. Briefly, tail warts were harvested and then homogenized with glass beads (catalog number G4649; Sigma) in phosphate-buffered saline (PBS) with 0.1 M NaCl by using a minibeadbeater (BioSpec Products). The wart extract was centrifuged to remove tissue, and the supernatant was collected and stored at −20°C for future infectious challenge. To quantify genome copy numbers in wart extracts, DNA was extracted with a Quick-DNA Miniprep Plus kit (catalog number D4068; Zymo Research) and amplified via reverse transcription-quantitative PCR (qRT-PCR) using primers 5′-AGAGTGCATGGCTGGCAAGA-3′ and 5′-CATGTGGCGCACCAAGTGAA-3′ and probe 5′-FAM (6-carboxyfluorescein)-TGGCAAGCCGCACGCTTTGGCATCA-TAMRA (6-carboxymethyltetrarhodamine)-3′. The reactions were carried out with 10-μl samples using Universal master mix (Applied Biosystems), 500 nM the forward and reverse type-specific primers, 250 nM double-labeled probe, and 2 μl of template DNA. Amplification was performed with a 7500 real-time PCR system (Applied Biosystems). The qPCR conditions were as follows: DNA polymerase activation at 95°C for 10 min was followed by 40 cycles consisting of denaturation for 15 s at 95°C and hybridization of primers and the probe as well as DNA synthesis for 1 min at 60°C. To produce a standard curve, religated MmuPV1 genomic DNA was serially diluted in nuclease-free reagent-grade water. The Applied Biosystems default settings for the threshold cycle (*C_T_*) were used for data analysis. The *C_T_* values were plotted against the copy number to generate a standard curve, which displayed an *R*^2^ value of 0.97 and a slope of −2.96. The assay showed linearity from 6 to 6 × 10^6^ copies/reaction.

Initial trauma for infection challenge of SKH-1 mice was done by using 2 to 5 passes of a Dremel rotary drill (setting 2) until the epidermis was disturbed. Twenty microliters of the wart (1.5 × 10^8^ MmuPV genome copies) supernatant was applied, followed by additional trauma by swabbing with endocervical brushes (catalog number 892010C500; Andwin Scientific) and, finally, the application of an additional 20 μl of the wart supernatant. All infection challenges were accompanied by challenge of athymic nude mice as a positive control to ensure the viability of the wart extract and effective challenge.

### DNA vaccination via electroporation.

The DNA vaccines targeting MmuPV1 and HPV16 were generated by direct synthesis (GenScript). The DNA vaccine (15 μg of DNA in 20 μl PBS) was intramuscularly injected into the hind leg thigh muscle, immediately followed by electroporation with a pair of electrode needles inserted into the muscle area surrounding the vaccine injection site. Electrical pulses were administered by using a BTX electroporation generator (catalog number ECM830; BTX Harvard Apparatus). Eight pulses of 106 V were delivered with a 20-ms pulse at 200-ms intervals.

### Chromogenic *in situ* hybridization.

Custom RNA *in situ* hybridization probes were developed to detect the full-length *E6*/*E7* mRNA sequence of MmuPV1 by Advanced Cell Diagnostics for use with their RNAscope assay kit. CISH was performed according to the manufacturer's instructions for RNAscope2.0 as previously described (37). Briefly, formalin-fixed, paraffin-embedded (FFPE) mouse tail tissue sections were pretreated with heat and protease prior to hybridization with the probe. If treated with RNase A (Qiagen), slides were incubated for 30 min at room temperature with RNase A in PBS (10 mg/ml). Slides were subsequently washed three times in diethyl pyrocarbonate (DEPC)-treated water for 5 min per wash. To ensure RNA integrity and assay procedures, adjacent sections were also hybridized with a probe for the endogenous housekeeping gene *ubiquitin* and the bacterial gene *dapB* (negative control). After washing, a horseradish peroxidase (HRP)-based amplification system was then used to detect the target probes, followed by color development with 3,3′-diaminobenzidine (DAB).

### mE6 and mE7 peptides.

A panel of 20-mer peptides, each overlapping by 15 amino acids, was generated by GenScript Inc. at a purity of ≥70% for both MmuPV1 E6 (25 peptides) and E7 (20 peptides). These 20-mers were then pooled into libraries of 5 peptides covering amino acids 1 to 40 (p1), 25 to 65 (p2), 50 to 90 (p3), 75 to 115 (p4), and 100 to 140 (p5) for E6 and amino acids 1 to 40 (p1), 25 to 65 (p2), 50 to 90 (p3), and 75 to 110 (p4) for E7. The 9-mer peptides, overlapping by 1 amino acid, for E6 amino acids 89 to 104 and E7 amino acids 76 to 95 were synthesized to further define their CD8^+^ T cell epitopes.

### Epitope mapping by intracellular cytokine staining.

Splenocytes were harvested from mice and immediately ground through 70-μm cell strainers (Falcon) into RPMI growth medium with 10% fetal bovine serum (FBS) (Gibco). Splenocytes were divided into 10^7^ cells/well and incubated with the above-described peptide libraries of MmuPV1 E6 or E7 and a Golgi plug for 12 h. Cells were stained by using fluorescein isothiocyanate (FITC)–anti-gamma interferon (IFN-γ) (catalog number 554411; BD Biosciences) and phycoerythrin (PE)–anti-CD8a (catalog number 553032; BD Biosciences) antibodies and collected for flow cytometry analysis.

### *In vivo* T cell depletion.

SKH-1 mice were administered 100 μg of monoclonal antibody to CD3 (clone 145-2C11; BioXcell) through intraperitoneal injection. Depletion was initiated by three daily injections followed by a once-weekly injection, which was maintained for 6 weeks.

### Enzyme-linked immunosorbent assay.

MmuPV1 E6, E7, and L2 serum antibody levels were measured by direct ELISAs. The MmuPV1 E6, E7, and L2 or HPV16 E6, E7, and L2 proteins were expressed as 6-His fusions by cloning between the EcoRI and HindIII sites in the pET-28a(+) vector and expressed in Escherichia coli. Proteins were then column purified by using Ni-nitrilotriacetic acid (NTA)-agarose (catalog number 30210; Qiagen) in 8 M urea. MmuPV1 L1 VLPs and HPV16 PsV were developed by using previously described protocols (82). For ELISA antigen binding, 500 ng of MmuPV1 E6, E7, L2, or PsV and 1 μg of HPV16 E6, E7, and L2 in PBS were incubated for 12 h on Nunc-Immuno MicroWell MaxiSorp plates (catalog number M9410; Sigma) at 4°C. Plates were blocked in 1% (wt/vol) bovine serum albumin (BSA)–PBS-T (PBS plus 0.01% [vol/vol] Tween 20) for 1 h at 37°C and subsequently incubated with 1:50-diluted sera in PBS-T. Following serum incubation for 1 h at 37°C, plates were washed three times in PBS-T and incubated with secondary anti-mouse HRP-conjugated antibody (1:5,000; GE Healthcare) for 1 h at 37°C, followed by washing three times and development with a 2,2′-azinobis(3-ethylbenzthiazolinesulfonic acid) (ABTS) solution (catalog number 11684302001; Roche).

### Vaginal challenge study.

BALB/cJ mice 2 weeks after the final DNA vaccination (described above) or Gardasil-9 (1/10 the human dose) vaccination were used for challenge. Mice were injected subcutaneously with 3 mg of medroxyprogesterone (Depo-Provera) and held for 4 days before vaginal challenge to synchronize their estrus cycles. Mice were vaginally challenged by using a mixture of 12 μl of HPV16 PsV and 28 μl of 3% carboxymethyl cellulose (CMC). Twenty microliters of the PsV-CMC mixture was injected into the vaginal tract before and after abrasion by an endocervical brush (25 rotations in alternating directions) (catalog number 892010C500; Andwin Scientific) while mice were anesthetized. Bioluminescence imaging to measure HPV16 PsV infection was conducted with a Xenogen IVIS 200 instrument (Caliper) 3 days after challenge, and images were acquired for 10 min. Region of interest (ROI) analysis was done by using Living Image 2.0 software.

### Western blot analysis.

293TT cells were transfected by using TransIT-202 transfection reagent (Mirus), using 2.5 μg pNGVL4a-hCRT-E6E7L2 (lot number 35266A) (28) DNA/well in 6-well plates, according to the manufacturer's guidelines. The cell lysate was made by using M-Per mammalian protein extraction reagent (catalog number 78501; Thermo Fisher) 48 h later, and the amount of protein was quantified by a bicinchoninic acid (BCA) assay (catalog number 23225; Thermo Pierce). Primary antibodies used were mouse monoclonal antibody RG1.1 to HPV16 L2 residues 17 to 36 for L2 detection, anti-HPV16 E6 clone E6-6F4 (Euromedex), and anti-HPV16 E7 clone 8C9 (catalog number 28-0006; Thermo Fisher). Blots were visualized by using ECL anti-mouse HRP-linked secondary antibody (catalog number NA931; GE Healthcare) (1:10,000) and HyGLO Quick Spray chemiluminescent HRP antibody detection reagent (catalog number E2400; Denville).
